# Body Composition Analysis of 10 Years versus 5 Years of Adjuvant Endocrine Therapy in Patients with Nonmetastatic Breast Cancer

**DOI:** 10.1155/2021/6659680

**Published:** 2021-01-16

**Authors:** Ruyi Hu, Xinran Cheng, Jun Liu, Xu Lai, Ruifeng Wang, Dongchang Yu, Yanan Fan, Zhaoshi Yu

**Affiliations:** Thyroid Breast Surgery Department, Hubei Ezhou Central Hospital, Ezhou, Hubei, China

## Abstract

**Objective:**

Our study aims to investigate the association of extended adjuvant endocrine therapy with disease-free survival (DFS), muscle mass, muscle strength, and visceral adipose tissue in patients with nonmetastatic breast cancer and the effect of extended endocrine therapy on body composition. *Patients and Methods*. Patients (*N* = 90) with nonmetastatic breast cancer aged between 60 and 65 years old were prospectively recruited in this study, compromising a cohort of subjects rece iving 5 years or 10 years of adjuvant endocrine therapy. Patients' DFS was compared between these two groups. Patients' body composition including muscle and fat using CT scans, muscle strength, and gait speed was evaluated in these two groups.

**Results:**

Dietary behavior was recorded with the food frequency questionnaire (FFQ). Patients' age, body weight, and body mass index (BMI) did not differ between the two groups. An extended adjuvant endocrine therapy into 10 years could translate into DFS benefit (123.8 vs. 102.2 months, *P*=0.038). Patients receiving 10 years of adjuvant endocrine therapy had less skeletal muscle and more visceral fat compared with patients receiving 5 years of adjuvant endocrine therapy. The skeletal muscle index was 50.3 ± 1.6 cm^2^/m^2^ versus 46.5 ± 1.3 cm^2^/m^2^ in the 10 years or 5 years of adjuvant endocrine therapy group (*P*=0.042). The visceral fat was 28.9 ± 2.9 cm^2^/m^2^ versus 55.0 ± 3.2 cm^2^/m^2^ in the 10 years or 5 years of adjuvant endocrine therapy group (*P*=0.011). The muscle strength, gait speed, and FFQ results in the two groups not reaching statistical difference.

**Conclusion:**

In conclusion, breast cancer patients with 10 years of adjuvant endocrine therapy had DFS benefit, but with more muscle loss and adipose tissue deposits compared to patients receiving 5 years of adjuvant endocrine therapy.

## 1. Introduction

Breast cancer is the first most common malignancy in females worldwide [1]. The average 10-year survival rate for women with invasive breast cancer reaches 90% [2]. Early breast cancer patients with the chance of surgery have a better survival rate. Adjuvant endocrine therapy undoubtedly extends the survival time [3–5]. Patients with early breast cancer are suggested to take at least 5 years of adjuvant endocrine therapy [5]. However, there is still inconsistency regarding the length of adjuvant endocrine therapy. Some oncologists recommend patients to extend endocrine therapy to 10 years to control the increased recurrence rate and mortality after patients have taken 5 years of adjuvant therapy. However, the study conclusions are controversial although several studies have published the survival data of comparison of early breast cancer patients receiving 5 years of adjuvant endocrine therapy in comparison with patients receiving 10 years of adjuvant endocrine therapy [6]. A previous clinical study of 30848 patients with breast cancer suggested that more than an extended 10-year endocrine therapy yields a disease-free survival (DFS) benefit for patients with early-stage breast cancer. It is worth more clinical data about the survival benefits and adverse events before recommendations of extended adjuvant endocrine therapy were given.

The adverse events of endocrine therapy could be decreased muscle mass and increased fat tissue. Accelerated muscle loss, also called sarcopenia, has been proved to be correlated to the menopausal transition and thus linked to declining estrogen levels [7]. Estrogen may take part in muscle metabolism through estrogen receptors found on skeletal muscle tissues [8–10], together with indirectly function targeting at the somatotropic axis by altering secretions of both growth hormone and insulin-like growth factor [11, 12]. Estrogen directly affects the structure of musculoskeletal tissues, such as muscle and ligament [13]. Restoring normal estrogen levels helps to restore cellular redox and glucose homeostasis in skeletal muscle. Estrogen improves muscle mass and muscle strength. Women suffered from accelerated muscle loss and decrease in muscle strength and function when they enter menopause since women in the menopausal period have lower circulating levels of estrogen and progesterone [14]. Muscle mass has been proved to be increased with hormone replacement therapy in postmenopausal women, regardless of exercise [15]. As an estrogen-dependent tumor, breast cancer cells will accelerate growth under the action of estrogen. Adjuvant endocrine therapy, for early breast cancer patients, consists of taking drugs with the function of antagonists of estrogen and progesterone could stop the proliferation of breast cancer cells. A breast cancer mouse model study proved that aromatase inhibitors, as a blocker of estrogen biosynthesis used as standard endocrine therapy, cause muscle weakness and bone loss [16]. Prevention of aromatase inhibitors-induced osteoclastic bone resorption with the bisphosphonate could attenuate the prevalence of bone metastases and improve muscle function in the mouse model. Nevertheless, the function of estrogen on human skeletal muscle was not clearly stratified due to the impact of many confounding differences, such as age, quality of life, dietary behavior, and intensity of exercise. Several studies researched the progesterone effects on skeletal muscle mass and its function. Progesterone receptor is found to be present in skeletal muscle cells and some functions of this steroid hormone in skeletal muscle tissue are studied [17]. Previous studies proved that treatment with progesterone has no impact on the ability of the skeletal muscle to oxidize lipids [18]. However, the relationship between progesterone and skeletal muscle mass and strength in breast cancer patients is not clearly stated.

Previous studies proved that hormones significantly influence body visceral adipose tissue distribution and visceral adipose differentiation [19]. Fat tissue is the original source of many proinflammatory cytokines in patients with more adipose tissue. Estrogen and its receptors regulate various aspects of glucose together with lipid metabolism. The decrease of estrogen is common during the menopausal period with characteristics of lipid profile variations and predominant visceral fat accumulation. The absence of estrogen has a significant impact on obesity in the menopausal period in women. Increased levels of estrogen because of excessive aromatization activity of the fat tissue and adipokines derived from fat tissue contribute to the risk to develop into breast cancer in obese women [20]. Obesity-related estrogen is a single independent prognostic factor for breast cancer patients [20]. Patients' large breast volume is proportional to the visceral adipose tissue deposits in obese premenopausal patients [21]. In postmenopausal breast cancer patients, a high visceral fat area was significantly associated with shorter distant DFS [22]. The association of adipose tissue and breast cancer is correlated to the imbalance of hormones, that is, estrogen (estrone and estradiol) [23]. In a clinical study recruiting totally 16608 women, breast cancer incidence was higher in the estrogen plus progestin group and more commonly occurred in lymph node-positive patients [24]. Aromatase mediates the crosstalk of obesity-associated inflammation and hormone alterations in patients with breast cancer [25]. The association of extended adjuvant endocrine therapy with obesity for patients with nonmetastatic breast cancer is still not clearly stated.

Thus, our study aims to investigate the association of extended adjuvant endocrine therapy with DFS, muscle mass, muscle strength, and visceral adipose tissue in patients with nonmetastatic breast cancer. Our hypothesis is that 10 years of adjuvant endocrine therapy could prolong the DFS, but decrease the muscle mass and muscle strength and increase adipose tissue at the same time.

## 2. Patients and Methods

### 2.1. Study Patients

90 patients with nonmetastatic breast cancer aged between 60 and 65 years old were prospectively recruited in this study, compromising a cohort of subjects that receive at least 5 years of adjuvant endocrine therapy. Patients were recruited from July 2017 to March 2020. We selected patients with a random number table. Inclusion criteria are breast cancer patients receiving already 5 years or 10 years of adjuvant endocrine therapy, with age of 60–65 years. Patients were not subjected to radiotherapy. Exclusion criteria included patients with diabetes, metabolic syndrome, tuberculous, rheumatoid arthritis, gout, systematic inflammatory disease, cerebrovascular disease, liver or renal insufficiency, cardiac insufficiency, autoimmune disease, or other severe systemic diseases. All patients were not allowed to receive steroids prior to this study. The study was approved by the Ethics Committee of Hubei Ezhou Central Hospital. All patients signed the written informed consent.

### 2.2. Baseline Characteristics

We recorded patients' baseline clinical data, including age, weight, body mass index (BMI), skeletal muscle index, visceral adipose index, tumor size, lymphatic invasion, number of metastatic lymph nodes, estrogen receptor (ER) status, progesterone receptor (PR) status, human epidermal growth factor receptor 2 (Her-2) status, adjuvant endocrine therapy, previous chemotherapy, target therapy, and surgery method (Table 1).

### 2.3. Body Composition Analysis

**Figure 1:**
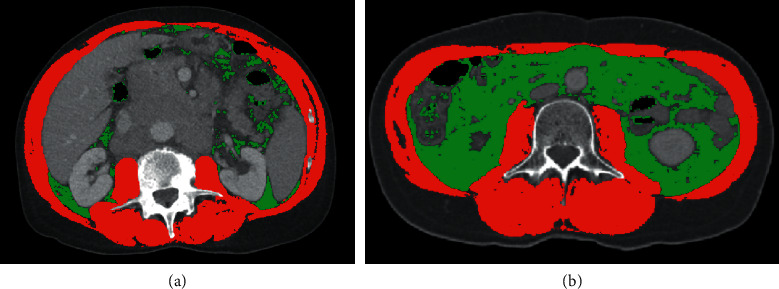
Example of body composition analysis using SliceOmatic software. The red zone represents muscle mass and green zone represents visceral adipose tissue. (a) Patients with low fat. (b) Patients with high fat.

We evaluated body composition including skeletal muscle mass and visceral adipose area at the third lumbar vertebrae (L3) level using abdominal CT scan using sliceOmatic software (version 5.0, Tomovision, Montreal, Canada) both at baseline and after 5 y or 10 y of endocrine treatment. The skeletal muscle was colored with typical Hounsfield units (HU) ranging from −29 to 150 and visceral adipose tissue was colored with HU ranging from −150 to −50 (Figure 1). The normalized skeletal muscle index and visceral adipose index were calculated by division of square of height. Skeletal muscle index = skeletal muscle mass/height^2^ (cm^2^/m^2^). Visceral adipose index = visceral adipose area/height^2^ (cm^2^/m^2^).

### 2.4. Muscle Strength and Gait Speed

Muscle strength was measured using handgrip strength. For females, handgrip strength less than 20 kg was regarded as abnormal [26]. A common gait speed test is called the 6-meter usual walking speed test. A cut-off of ≤0.8 m/s is regarded as abnormal by EWGSOP2 [26].

### 2.5. The Food Frequency Questionnaire (FFQ)

Patients' dietary behavior was assessed using the food frequency questionnaire (FFQ). Patients were asked to do a 24-hour dietary recall. Patients' energy intake, protein, fat, and carbohydrate intake were recorded.

### 2.6. Statistical Methods

We did all statistical analysis using SPSS 23 (IBM, Armonk, NY). Descriptive statistical analyses of baseline characteristics were conducted for all continuous variables by mean values (standard deviation) and categorical variables by numbers (percentage). Comparisons of skeletal muscle index, visceral adipose index, muscle strength, gait speed between patients with 5 years of adjuvant endocrine therapy and 10 years of adjuvant endocrine therapy were made using Student's *t* test with *P* < 0.05 considered as statistically significant. Patients' dietary behaviors between the two groups were also compared using Student's *t* test.

## 3. Results

### 3.1. Baseline Characteristics

Patients' age, body weight, and BMI did not differ between the two groups (Table 1). Patients baseline tumor size, lymphatic invasion, number of metastatic lymph nodes, estrogen receptor (ER)/progesterone receptor (PR) status, human epidermal growth factor receptor 2 (Her-2) status, endocrine therapy, and surgery are summarized in Table 1. The median age in 5 years of endocrine therapy group was 62.5 years versus 62.8 years in the 10 years of adjuvant endocrine therapy group (*P*=0.521). The average weight in 5 years of endocrine therapy group was 72.2 kg versus 72.6 kg in the 10 years of adjuvant endocrine therapy group (*P*=0.529). The average BMI in 5 years of endocrine therapy group was 26.1 kg/m^2^ versus 26.3 kg/m^2^ in the 10 years of adjuvant endocrine therapy group (*P*=0.809), both falling within the overweight range proposed by the World Health Organization.

### 3.2. Impact of 5 Years or 10 Years of Adjuvant Endocrine Therapy on DFS

**Figure 2:**
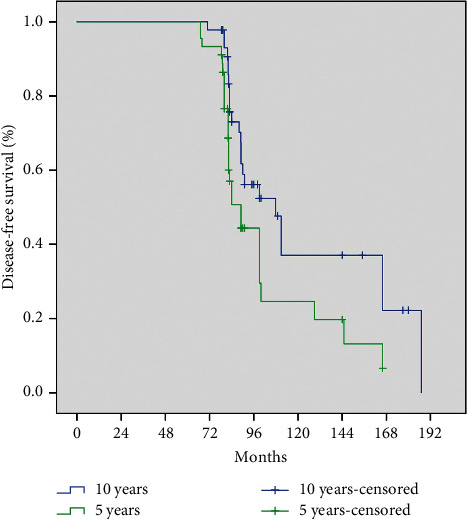
Kaplan-Meier curve of breast cancer patients receiving 5 years or 10 years of adjuvant endocrine therapy. Patients receiving 10 years of adjuvant endocrine therapy had better DFS compared to patients receiving 5 years of adjuvant endocrine therapy (123.8 vs. 102.2 months, *P*=0.038).

An extended adjuvant endocrine therapy into 10 years could translate into DFS benefit. Patients with 10 years of adjuvant endocrine therapy had better DFS compared with patients with 5 years of adjuvant endocrine therapy (123.8 vs. 102.2 months, *P*=0.038) (Figure 2).

### 3.3. Impact of 5 Years or 10 Years of Adjuvant Endocrine Therapy on Body Composition

**Figure 3:**
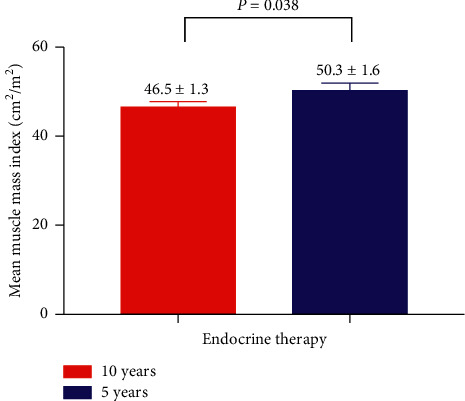
Comparison of muscle mass index of breast cancer patients receiving 5 years or 10 years of adjuvant endocrine therapy. Breast cancer patients receiving 5 years of adjuvant endocrine therapy had higher muscle index compared with breast cancer patients receiving 10 years of adjuvant endocrine therapy (50.3 ± 1.6 vs. 46.5 ± 1.3 cm^2^/m^2^, *P*=0.042).

**Figure 4:**
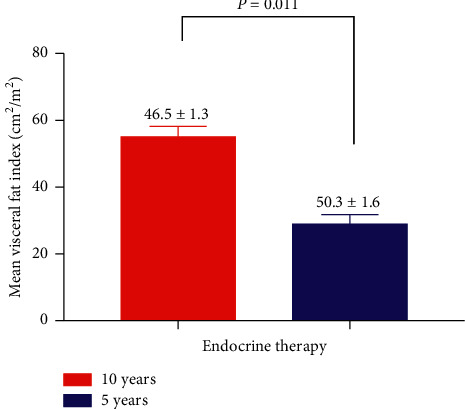
Comparison of visceral fat of breast cancer patients receiving 5 years or 10 years of adjuvant endocrine therapy. Breast cancer patients receiving 5 years of adjuvant endocrine therapy had less visceral fat compared with breast cancer patients receiving 10 years of adjuvant endocrine therapy (28.9 ± 2.9 vs. 55.0 ± 3.2 cm^2^/m^2^, *P*=0.011).

Patients' baseline skeletal muscle index or visceral fat did not have a statistical difference (Table 1). Breast cancer patients receiving 10 years of adjuvant endocrine therapy had less skeletal muscle and more visceral fat compared with patients receiving 5 years of adjuvant endocrine therapy. The skeletal muscle index in 10 years endocrine therapy group was 55.0 ± 3.2 cm^2^/m^2^ versus 28.9 ± 2.9 cm^2^/m^2^ in the 5 years of adjuvant endocrine therapy group (28.9 ± 2.9 vs. 55.0 ± 3.2 cm^2^/m^2^, *P*=0.042) (Figure 3). The visceral fat in 10 years endocrine therapy group was 28.9 ± 2.9 cm^2^/m^2^ versus 55.0 ± 3.2 cm^2^/m^2^ in the 5 years of adjuvant endocrine therapy group (28.9 ± 2.9 vs. 55.0 ± 3.2 cm^2^/m^2^, *P*=0.011) (Figure 4).

### 3.4. Impact of 5 Years or 10 Years of Adjuvant Endocrine Therapy on Muscle Strength and Gait Speed

Patients' muscle strength was defined by handgrip strength. The muscle strength in the two groups did not differ, which was 27.6 ± 2.3 kg in 5-year endocrine therapy group and 21.9 ± 1.5 kg in the 10-year endocrine therapy group (*P*=0.329) (Table 2). The gait speed of 6 m walking usual pace in two groups did not differ, which was 4.4 ± 0.1 s in 5-year endocrine therapy group and 4.6 ± 0.1 s in the 10-year endocrine therapy group (*P*=0.798) (Table 2).

### 3.5. Comparison of Patients Dietary Behaviors in 5 Years or 10 Years of Adjuvant Endocrine Therapy

Patients took a 24-hour dietary recall using the FFD (Table 3). Patients' energy intakes were 1942.1 ± 45.5 kcal in the 5-year endocrine therapy group versus 1973.1 ± 76.6 kcal in the 10-year endocrine therapy group (*P*=0.412). Patients' protein intakes were 65.1 ± 9.9 g in the 5-year endocrine therapy group versus 64.1 ± 9.7 g in the 10-year endocrine therapy group (*P*=0.221). Patients' fat intakes were 50.9 ± 5.2 g in the 5-year endocrine therapy group versus 48.7 ± 8.2 g in the 10-year endocrine therapy group (*P*=0.756). Patients' carbohydrate intakes were 296.7 ± 43.1 g in the 5-year endocrine therapy group versus 301.1 ± 31.8 g in the 10-year endocrine therapy group (*P*=0.109).

## 4. Discussion

Our study was the first to evaluate the impact of extended endocrine therapy on patients' DFS, body composition, muscle strength, and gait speed. We proved that patients receiving 10 years of endocrine had DFS benefit, which is in accordance with a previous study [27]. However, this 10-year therapy could lead to patients' decline in muscle mass and raise in visceral adipose even though no significant difference in muscle strength and gait speed were observed. Our patients also did a FFQ, which could rule out the impact of dietary on patients' muscle and fat.

Muscle maintenance is a very important issue for cancer patients. Numerous studies have indicated that cancer patients with muscle loss had worse survival [28–32]. In patients with stages I–III of colorectal cancer, muscle wasting is an independent risk factor for all-cause mortality, regardless of other body composition parameters [28]. In a retrospective study of 2042 patients undergoing radical prostatectomy of prostate cancer, low muscle mass was approved to be associated with increased risks of recurrence and mortality, no matter the BMI [30]. A retrospective study of 231 metastatic gastric cancer patients proved that skeletal muscle loss during chemotherapy was associated with shorter overall survival (OS) [31]. Over one-third of newly diagnosed patients with nonmetastatic breast cancer patients were found to have low muscle radiodensity [32]. In an observational study of 3241 patients diagnosed with nonmetastatic breast cancer, low muscle radiodensity and high total adipose tissue were statistically significantly related to overall mortality [32].

Adipose tissue contributes to cancer development and is negatively associated with prognosis in several cancer types [33–37]. There was an association between obesity and breast cancer risk in postmenopausal ER/PR-positive patients in many clinical studies [35–37]. Obesity was associated with shorter DFS and increased mortality rate in both premenopausal and postmenopausal breast cancer patients. Women who had a BMI higher than 40 kg/m^2^ had a 2-fold higher risk of mortality rate in comparison with breast cancer patients with normal BMI (RR, 2.12) [38]. Other studies proved that higher adipose tissue was associated with shorter distant DFS and OS, larger tumors, positive lymph node status, and triple-negative tumor subtype [39, 40].

Estrogen and progesterone are the two primary female sex hormones. They are produced mainly in ovaries and produced or converted into forms of estrogen in fat tissue. Estrogen balance is essential for achieving and maintaining muscle and fat loss. Estrogen significantly facilitates adipose tissue depots and its function [41]. Estrogen protects against accumulated visceral adipose through its function to suppress appetite and raise energy expenditure. A clinical study of 294 patients with invasive breast cancer proved that invasive breast cancer mainly occurs adjacent to breast adipose tissue, irrespective of the tumor volume and patients' ER/PR status [42]. A previous trial ATLAS proved that patients treated with tamoxifen for ten years have reduced risk of breast cancer recurrence and mortality compared to those treated with tamoxifen for only 5 years [43]. However, more side effects occurred, such as loss of bone mineral density [44]. There is no published article regarding the impact of extended adjuvant endocrine therapy on body composition, muscle strength, and gait speed. Our study was the first to prove that 10 years of adjuvant could decrease patients' muscle mass index and increase their adipose tissue even though they had almost the same daily energy intakes compared with patients with 5 years of adjuvant therapy.

Our study also has limitations. Our sample size is not large, which means the conclusions still need to be validated in a larger-scale clinical trial. Our study is an observational cohort prospective study; thus, we could not rule out confounding factors that could impact muscle or fat, such as exercise. However, we tried our best to do a FFQ, which rule out the impact of dietary on muscle and fat. Patients' baseline characteristics including age, sex, and BMI did not reach significant difference. However, patients with the same BMI may have different adipose tissue compartments and muscle mass. Our study was the first to look further into the impact of endocrine therapy on body composition in breast cancer patients.

## 5. Conclusion

In conclusion, breast cancer patients with 10 years of adjuvant endocrine therapy had better DFS, but with more muscle loss and adipose tissue deposits compared with patients receiving 5 years of adjuvant endocrine therapy.

## Figures and Tables

**Table 1:** Patients baseline characteristics.

	Patients with 5 years of adjuvant endocrine therapy (*n* = 45)	Patients with 10 years of adjuvant endocrine therapy (*n* = 45)	*P* value
Age (median)	62.5	62.8	0.521

Weight (kg)	72.2	72.6	0.529

BMI (kg/m^2^)	26.1	26.3	0.809

Muscle mass index (cm/m^2^)	61.5	64.1	0.217

Visceral adipose index (cm/m^2^)	25.1	26.9	0.176

Tumor size			
T1	37	32	
T2	8	13	

Lymphatic invasion			
N0	35	33	
N1	10	12	

Number of metastatic lymph nodes			
M0	45	45	

ER/PE status			
ER+/PR+	30	27	
ER+/PR−	9	6	
ER−/PR+	6	7	
ER−/PR−	0	5	

Her-2 status			
Positive	15	23	
Negative	30	22	

Adjuvant endocrine therapy			
Anastrozole	8	7	
Letrozole	13	16	
Tamoxifen	12	10	
Exemestane	12	12	

Previous chemotherapy			
Doxorubicin + cyclophosphamide	19	18	
Docetaxel + cyclophosphamide	19	22	
Docetaxel + carboplatin	7	5	

Target therapy (transtuzumab)			
Yes	15	21	
No	30	24	

Surgery			
Modified radical mastectomy	11	13	
Breast conservation	34	32	

BMI: body mass index; ER: estrogen receptor; PR: progesterone receptor; Her-2: human epidermal growth factor receptor 2.

**Table 2:** Patients muscle strength and gait speed in the two groups.

Mean ± standard deviation	Patients with 5 years of adjuvant endocrine therapy (*n* = 45)	Patients with 10 years of adjuvant endocrine therapy (*n* = 45)	*P* value
Muscle strength (kg)	27.6 ± 2.3	21.9 ± 1.5	0.329
Gait speed of 6 m walking usual pace (s)	4.4 ± 0.1	4.6 ± 0.1	0.798

**Table 3:** Patients dietary behaviors in two groups.

	Patients with 5 years of adjuvant endocrine therapy (*n* = 45)	Patients with 10 years of adjuvant endocrine therapy (*n* = 45)	*P* value
Energy (kcal)	1942.1 ± 45.5	1973.1 ± 76.6	0.412
Protein (g)	65.1 ± 9.9	64.1 ± 9.7	0.221
Fat (g)	50.9 ± 5.2	48.7 ± 8.2	0.756
Carbohydrate(g)	296.7 ± 43.1	301.1 ± 31.8	0.109

## Data Availability

The datasets analyzed during the present study are available from the corresponding author on reasonable request.
